# Zellweger’s Syndrome With PEX6 Gene Mutation in Mixteco Neonates Due to Possible Founder Effect

**DOI:** 10.7759/cureus.45162

**Published:** 2023-09-13

**Authors:** Daniel Slaton, Ashley Chang, Tamanna Ahluwalia, Sophie Alfaro, Britani Javed, Rocky Greer

**Affiliations:** 1 School of Osteopathic Medicine, Andrew Taylor (AT) Still University, Mesa, USA; 2 Clinical Science Education, Andrew Taylor (AT) Still University, Mesa, USA; 3 Neonatal Medicine, Marian Regional Medical Center, Santa Maria, USA

**Keywords:** neonatal mortality, neonatal screening, peroxisomal disorders, inherited metabolic diseases, consanguinity marriage, founder mutation, mixteco, pex6, zellweger disease

## Abstract

Zellweger spectrum disorder (ZSD) is a group of autosomal recessive peroxisomal disorders caused by *PEX* gene mutations that commonly present with symptoms of severe hypotonia, epileptic seizures, failure to thrive, hepatomegaly, craniofacial dysmorphisms, and sensorineural hearing loss. This article highlights three patients born with ZSD in Central California. All three patients were born to Mixteco mothers. Patients were genetically analyzed, which revealed mutations that correspond to ZSD. They presented with hypotonia at birth, abnormal hepatic panels, and increased fatty acid levels, findings consistent with Zellweger syndrome (ZS). However, only two of three patients displayed sensorineural hearing loss. Two of the patients failed to survive more than one year of age, which reflects the average life expectancy of an infant presenting with ZS. Observed and recorded cases of ZS in the Mixteco population have been postulated to be related to consanguinity and/or a founder effect. Studies have shown that autosomal recessive diseases are more prevalent in consanguineous populations. Consanguinity has been denied by patient 1 and is unknown for patients 2 and 3. Founder mutations have been implicated in areas with high rates of autosomal recessive diseases. All three of our Mixteco patients share a distinct lineage as well as a mutation at *PEX6*, leading us to believe that they suffered from an inherited founder mutation. The Mixteco population is not studied well enough to come to a definitive conclusion; however, the recognition of the relationship between ZS and Mixteco background is important, as it allows parents to plan accordingly and increases awareness in the community.

## Introduction

Zellweger spectrum disorder (ZSD) comprises a wide spectrum of disease presentations that are caused by errors in peroxisomal biogenesis. These errors are mitigated by distinct autosomal recessive PEX gene mutations, which normally code for essential peroxisome structures. Peroxisomes are involved in phospholipid and bile acid biogenesis and matrix protein import. The dysfunction of either results in an abnormal accumulation of toxic metabolites such as very long-chain fatty acids (VLCFAs), phytanic and pristanic acid, and C27-bile acid intermediates. There are 16 PEX genes identified in humans, and pathogenic variants resulting in ZSD have been reported in PEX1, PEX2, PEX3, PEX5, PEX6, PEX10, PEX11β, PEX12, PEX13, PEX14, PEX16, PEX19, and PEX26. While the spectrum of disease severity is variable, with some patients living into adulthood, patients with neonatal presentations typically have a life expectancy of less than one year [1].

ZSD may be divided into three subgroups based on the age of presentation: the neonatal-infantile presentation, the childhood presentation, and the adolescent-adult (late) presentation [2]. The neonatal-infantile presentation, which is the presentation our patients were afflicted by, includes the following clinical features: severe hypotonia, epileptic seizures, failure to thrive, hepatomegaly, craniofacial dysmorphisms, and sensorineural hearing loss. Diagnostic tests also often show elevated liver enzymes, low APGAR scores, and failed auditory brainstem response (ABR) testing. There is no curative treatment for ZSD, but symptomatic therapies such as Lorenzo’s oil and cholic acid therapy have been the subject of recent experimentation, with the latter having shown promising results in a 2016 study [3].

The incidence of ZSD is estimated to be 1/50,000 newborns in the United States [1]. We report three patients of Mixteco heritage who presented with ZSD to the same hospital. Given the rare nature of the disease, it is important to take into account a possible founder mutation as a cause for the observed cases of ZSD in the Mixteco population. It is also imperative to note that this population is especially understudied in empirical research. This is likely due to language, cultural barriers, and health disparities. While 60% of reported ZSDs are due to PEX1 gene mutations, all three of the patients discussed in this report were found to be homozygous for the PEX6 gene mutation. This correlates with the PEX6 gene founder mutation in patients with Zellweger's syndrome in the French-Canadian population. This may have implications for carrier screening and prenatal care for future mothers in this community. Given this suspected increased incidence of ZS in the Mixteco population, additional and more culturally inclusive research is imperative to detail the molecular basis of this occurrence and identify a putative founder mutation.

## Case presentation

Patient 1

A female was born at 41 weeks to a 21-year-old G2P1T1 Mixteco mother via vaginal delivery complicated by late decelerations and a tight nuchal cord. APGAR scores were 3 at 1 minute and 6 at 5 and 10 minutes (Table 1).

**Table 1:** Growth progress and APGAR score Weight, length, head circumference, and APGAR score at birth and discharge compared among three different patients. Decrease in weight, length, and head circumference noted for patients 1 and 2. Numbers in parenthesis correspond to the percentile ranking for that specific measurement.

	Patient 1	Patient 2	Patient 3
Birth weight	2.915 kg (5.8%)	3.750 kg (62.6%)	2.315 kg
Discharge weight	2.93 kg (1.0%)	3.500 kg (3.6%)	-
Birth length	51.5 cm (43.6%)	52.0 cm (65.5%)	47.0 cm
Discharge length	52.0 cm (16.1%)	53.0 cm (11.9%)	-
Birth head circumference	32.5 cm (2.6%)	34.3 cm (29.1%)	30.0 cm
Discharge head circumference	33.5 cm (0.7%)	36.0 cm (6.1%)	-
APGAR score at 1 min, 5 min, 10 min	3, 6, 6	1, 4, 5	7, 7, -

On examination, measurements were as follows: weight 2.915 kg (5.8%), length 51.5 cm (43.6%), and head circumference 32.5 cm (2.6%) (Table 1). The patient was symmetrically small for gestational age and presented with multiple congenital anomalies such as persistent pupillary membrane, dysmorphic facies, nocturnal lagophthalmos, microcephaly, and large fontanelles. She also exhibited an absence of bilateral red reflex, nonreactive unequal light reflex, microtia, exophthalmos, a high arched palate, redundant neck skin, as well as a large belly and inverted feet. Further investigation revealed hexacosanoic acid (C26:0) at 7.58 µmol/L, docosanoic acid (C22:0) at 12.79 µmol/L, and tetracosanoic acid (C24:0) at 22.27 µmol/L, as well as elevated C26/C22 and C24/C22 ratios, which are consistent with peroxisomal function disorder (Table 2).

**Table 2:** Long chain fatty acids and branched chain fatty acids Long chain fatty acid levels, long chain fatty acid ratios, and phytanic and pristanic acid levels compared among three different patients. Docosanoic acid level was low in all three patients. Hexacosanoic acid level, as well as ratios of tetracosenoic and hexacosanoic acid to docosanoic acid (C24/C22 and C26/C22, respectively), were high in all three patients. Phytanic acid and pristanic acid levels were normal for all three patients. Reference values are in parenthesis.

	Patient 1	Patient 2	Patient 3
Docosanoic acid (C22:0)	↓ 12.79 µmol/L (28.94–93.50)	↓ 19.51 µmol/L (28.94–93.50)	↓ 13.44 µmol/L (28.94–93.50)
Tetracosenoic acid (C24:0)	↓ 22.27 µmol/L (24.25–77.75)	39.18 µmol/L (24.25–77.75)	26.90 µmol/L (24.25–77.75)
Hexacosanoic acid (C26:0)	↑ 7.58 µmol/L (0.17–0.73)	↑ 8.27 µmol/L (0.17–0.73)	↑ 7.17 µmol/L (0.17–0.73)
C24/C22 ratio	↑ 1.74 (0.64–1.02)	↑ 2.01 (0.64–1.02)	↑ 2.00 (0.64–1.02)
C26/C22 ratio	↑ 0.592 (0.003–0.015)	↑ 0.424 (0.003–0.015)	↑ 0.534 (0.003–0.015)
Phytanic acid	<0.10 µmol/L (0.03–2.13)	0.38 µmol/L (0.03–2.13)	<0.10 µmol/L (0.03–2.13)
Pristanic acid	<0.10 µmol/L (0.00–0.31)	<0.10 µmol/L (0.00–0.31)	<0.10 µmol/L (0.00–0.31)

Laboratory evaluation revealed normal electrolytes, blood urea nitrogen (BUN), and creatinine levels. The hepatic panel was as follows: total bilirubin 1.7 mg/dL, direct bilirubin 2.5 mg/dL, aspartate transaminase (AST) 557 units/L, alanine transaminase (ALT) 215 units/L, gamma-glutamyltransferase (GGT) 120 units/L, and alkaline phosphatases 430 units/L (Table 3).

**Table 3:** Hepatic panel Highest levels of liver enzymes and glucose compared among three different patients. ALT (alanine transaminase), AST (aspartate transaminase), total bilirubin, and direct bilirubin were elevated in all three patients. Two out of three patients had elevated GGT (gamma-glutamyltransferase), alkaline phosphatase and glucose levels. Reference values are in parenthesis.

	Patient 1	Patient 2	Patient 3
ALT	↑ 215 units/L (0–55)	↑ 313 units/L (0–55)	↑ 60 units/L (0–55)
AST	↑ 557 units/L (5–34)	↑ 772 units/L (5–34)	↑ 246 units/L (5–34)
Total bilirubin	↑ 1.7 mg/dL (0.0–1.2)	7.0 mg/dL (0.0–10.0)	↑ 3.1 mg/dL (0.0–1.2)
Direct bilirubin	↑ 2.5 mg/dL (0.0–0.5)	↑ 5.5 mg/dL (0.0–0.5)	↑ 1.3 mg/dL (0.0–0.5)
GGT	↑ 120 units/L (12–64)	↑ 228 units/L (12–64)	-
Alkaline phosphatase	430 units/L (0–500)	↑ 910 units/L (0–500)	↑ 734 units/L (0–500)
Glucose	↑ 99 (50–80)	79 mg/dL (51–159)	↑ 102 mg/dL (60–99)

She presented with jaundice on day one, which resolved in a couple of weeks. She also presented with respiratory insufficiency. Initially, her respiratory insufficiency was believed to be secondary to transient tachypnea of the newborn (TTN). Later, it was hypothesized to be secondary to hypotonia, respiratory muscle weakness, and inadequate respiratory drive. On day 2, the patient was taken off of total parenteral nutrition (TPN) and feeds were started. In addition, the patient had an abnormal eye exam consistent with Axenfeld-Reiger syndrome, an autosomal dominant disorder marked by systemic developmental abnormalities secondary to neural crest cell differentiation/migration. The patient was positive for adrenoleukodystrophy (ALD) without the ABCD1 mutation. The presence of positive alpha-glucosidase and alpha-L-iduronidase enzyme activity ruled out lysosomal acid alpha-glucosidase deficiency (Pompe disease) and mucopolysaccharidosis I disorder.

An X-ray of the chest showed a mildly enlarged cardiac silhouette, consistent with TTN and cardiomegaly. Ultrasound (US) demonstrated a large atrial septal defect and patent ductus arteriosus with a bi-directional shunt, along with normal liver, gallbladder, and biliary findings. A brain ultrasound showed small bilateral third ventricle cysts. Genetic testing confirmed a pathogenic homozygous variant of undetermined significance in PEX6 c.1409G>C (p.Gly470Ala), which was confirmatory for ZS. The patient was discharged with the following: weight 2.930 kg (1.0%), length 52.0 cm (16.1%), and head circumference 33.5 cm (0.7%) (Table 1). Parents opted for do-not-resuscitate (DNR) orders and comfort care.

One month following discharge, the patient presented to the emergency department (ED) with diarrhea and was diagnosed with gas pain and an acute upper respiratory infection. Comfort measures were taken, and the patient was discharged the same day.

Patient 2

A male was born full-term to a 22-year-old G1P0 Mixteco mother via cesarean section. Pregnancy was complicated by pre-eclampsia, late prenatal care, and maternal fever. APGAR scores were 1, 4, and 5 at 1 minute, 5 minutes, and 10 minutes, respectively. On examination, the measurements were as follows: weight of 3.750 kg (62.6%), length of 52.0 cm (65.5%), and head circumference of 34.3 cm (29.1%) (Table 1). The patient presented with a large anterior fontanelle, soft and flat, an open posterior fontanelle, upslanting palpebral fissures, almond-shaped eyes, hypertelorism, significant cranial molding, hypotonia, and a single palmar crease. Karyotyping revealed 46 and XY, ruling out trisomy 21. Initial laboratory tests revealed normal electrolytes, BUN, and creatinine levels. The hepatic panel was as follows: total bilirubin 3.2 mg/dL, direct bilirubin 1.9 mg/dL, AST 544 units/L, ALT 268 units/L, GGT 228 units/L, and alkaline phosphatases 481 units/L. Repeat laboratory tests revealed the following: total bilirubin 7.0 mg/dL, direct bilirubin 5.5 mg/dL, AST 772 units/L, ALT 313 units/L, GGT 228 units/L, and alkaline phosphatase 910 units/L (Table 3). He presented with jaundice on day 1, which resolved in one week. He developed signs of respiratory insufficiency with intermittent tachypnea, inspiratory stridor, tracheal tugging, and laryngomalacia on day 15. He presented with poor tolerance when feeding and transitioned from gavage to oral on day 30. The patient failed a bilateral ABR hearing test.

Further investigation revealed the following: C26:0 8.278 umol/L, C22:0 19.51 µmol/L, and elevated C26/C22 and C24/C22 ratios, consistent with peroxisomal function disorder (Table 2). Low red blood cell plasmalogen/fatty acid ratios were consistent with a defect in plasmalogen metabolism. The patient was positive for ALD without the ABCD1 mutation. Pompe disease and mucopolysaccharidosis I disorder were ruled out in the presence of positive alpha-glucosidase and alpha-L-iduronidase enzyme activity.

US revealed a normal liver, grade one bilateral hydronephrosis, and bilateral cysts around the lateral ventricles. ZS was finally confirmed by genetic testing, which revealed an autosomal homozygous pathogenic variant of the PEX6 gene c.1409G>C (p.Gly470Ala).

He was discharged with the following parameters: weight 3.500 kg (3.6%), length 53.0 cm (11.9%), and head circumference 36.0 cm (6.1%) (Table 1). Three months later, he was brought to the ED by his parents to pass out in the hospital. He appeared cachectic, had agonal respiration, sunken eyes, and gray skin. His capillary refill was >2 seconds, and he was tachycardic. His weight was 3.3 kg, his height was 58 cm, and his body mass index was 9.81 kg/m2. The patient has since passed.

Patient 3

A female was born full-term to a G2P1 23-year-old female of Mixteco heritage through normal vaginal delivery. Consanguinity status was unknown. Family history was significant for a brother who had the same condition and later passed away at five months of age. Prenatal genetic testing was positive for a homozygous PEX6 mutation, confirming the suspected diagnosis of ZS. Screening was also positive for ALD.

At birth, the patient weighed 2.315 kg, was 47 cm tall, and had a head circumference of 30 cm (Table 1). Physical examination revealed a microcephalic infant with soft and large anterior fontanelles, slightly dysmorphic features, severe hypotonia at all four extremities, blunted reflexes, and high-pitched crying. Initial laboratory testing yielded elevated C26:0 levels of 7.17 µmol/L and elevated ratios of C26/22 and C24/22, consistent with a peroxisomal biogenesis disorder (Table 2). The following values were also elevated: total bilirubin (3.1 mg/dL), direct bilirubin (1.3 mg/dL), alkaline phosphatase (734 units/L), ALT (60 units/L), AST (246 units/L), and glucose (102 mg/dL) (Table 3). The patient also failed ABR testing twice.

Due to the known diagnosis, the parents opted for DNR and comfort care, and the patient was discharged the following day. However, the patient returned two months later to the ED with congestion, a cough, and a fever. This led to a 12-day hospital course due to an infection with the influenza A virus that was complicated by bacterial pneumonia. One month later, the patient returned to the hospital. She was diagnosed with bilateral pneumonia. She was discharged to home hospice care but presented again to urgent care one month later for cough, congestion, and severe hypoxia. A physical exam at this visit showed severe hypotonia, a poorly defined skull, positional plagiocephaly, short palpebral fissures with shallow orbits, a short philtrum, and a thin upper lip.

The patient's date of death is unknown. Follow-up calls to the mother revealed the patient had passed, making her approximately six months old at the time of her passing.

## Discussion

Zellweger spectrum disorder is a group of peroxisomal biogenesis disorders with varying degrees of presentation. Commonly, ZSD leads to impaired α- and ß-oxidation of VLCFA, as well as impaired synthesis of bile acids. This results in increased levels of toxic bile acid intermediates that inevitably lead to multisystem organ damage. Table 2 demonstrates the lab values consistent with this diagnosis. All three of our patients were found to have elevated levels of C26:0 and C24:0, as well as elevated ratios of C26:0 to C22:0 and C24:0 to C22:0. Phytanic and pristanic acids are markers of peroxisomal metabolism and were normal in our patients. However, these levels would have presumably risen as the baby’s dietary intake increased [4,5].

The severity of ZSD depends on the time of presentation. Our patients were afflicted by the neonatal presentation of the disease, which is associated with a severe phenotype [1]. They presented with facial dysmorphisms, hypotonia, poor feeding, sensorineural deafness, and retinal dysfunction. In addition, patients 2 and 3 both failed the ABR test. These exam findings are all consistent with a ZSD diagnosis [1,4]. Our patients displayed elevated levels of liver enzymes consistent with hepatic dysfunction, which is also accordant with neonatal ZSD (Table 3) [4,6]. Further genetic testing revealed that two of these infants had the same pathogenic PEX6 genetic variant (c.1409G>C (p.Gly470Ala)), and all three had mutations in PEX6 [5]. Death in patients afflicted with neonatal ZSD often occurs within the first year of life. This occurred to patients 2 and 3 [1].

As aforementioned, the incidence of ZSD is rare in most populations (1/50,000 births in the United States). However, certain populations tend to exhibit greater disparities in incidence. There are a variety of explanations for these findings, including the presence of a founder mutation or consanguinity. For example, the incidence of ZSD in the French-Canadian population in the Saguenay-Lac-St-Jean region of Quebec (1/12,191) [7] and the Bedouin-Muslim population in Israel (4.6/100,000) has been attributed to a founder mutation and consanguinity, respectively [8]. The patients that we report on are of the Mixteco population, who have immigrated from Oaxaca, Mexico. Most of these Mixteco immigrants have taken up residence in Central California. Within the past few years, there have been several reported cases of the severe form of ZSD in this specific population. Six patients in particular were homozygous for the PEX6 novel variant c.1409G>C (p.Gly470Ala), which is the same variant displayed by two of our patients [7,9-10]. One of the theories proposed for this occurrence is based on consanguineous relationships within the Mixteco population. Studies on consanguineous relations and autosomal recessive inherited immunodeficiencies confirmed that these diseases are more prevalent in consanguineous populations [10]. Recessive and multifactorial disorders have been found to have higher values of consanguinity when compared to other genetic diseases [11]. For example, in southern Israel, the incidence of inherited metabolic disorders (IMDs) was found to be higher in offspring conceived from consanguineous versus non-consanguineous relations. Two siblings from a consanguineous Moroccan family developed moderate ZSD due to homozygous inheritance of an abnormal PEX1 gene [12]. Despite this, there have been no reports of consanguineous relations among Mixteco patients who gave birth to offspring with ZSD. In addition, Mixteco respondents reported an overall negative attitude towards cousin marriages [13]. It should be noted that consanguinity status was unknown or negative for each of our patient's parents. However, we did find one report of parental consanguinity in a Mixteco patient afflicted with ZSD [9].

Another explanation for the observed cases of ZSD in the Mixteco population is the founder effect. This occurs when genetic mutations occur at high frequencies as they are passed down from one or more ancestors within an isolated group. In the Saguenay-Lac-St-Jean region, a single PEX6 founder mutation was found to be responsible for the increased incidence of ZSD in that area. Although consanguinity is found in patients afflicted with a founder mutation, it is not required. Pedigrees from the Saguenay-Lac-St-Jean region denied any close family relationships that could otherwise explain the observed homozygosity for the mutation [7]. In Japan, analysis of homozygous two-base pair deletions in PEX10 was suggestive of a founder effect that led to the high frequency of ZSD within that population [6]. Individuals from indigenous populations in Oaxaca (including Mixteco populations) were genetically characterized. Results showed that the populations constituted one of two major genetic groups. This indicated that, despite geography and linguistics, these groups are genetically similar. This may imply that other individuals from these populations may be suffering from a founder effect and, therefore, an increased incidence of ZSD [14].

Our patients' presentations align with some of the same characteristics that are found in cases afflicted by founder mutations. Namely, a large number of Mixteco patients reported to have ZSD also have the same pathogenic PEX6 mutation. In addition, the fact that these patients share a distinct Mixteco lineage is also suggestive of a founder mutation [7,9].

Treatment of these patients is focused on optimizing quality of life. Specifically, children who suffer from ZSD need a special diet and help with oral feeding. Some patients may suffer from sensory hearing impairment and may benefit from hearing aids. Patients should be monitored for oxaluria, which can lead to renal complications. Seizures may be managed with anticonvulsants. The cause of death is usually due to respiratory or infectious complications. Patients suffering from cholestasis and hepatic injuries can be treated with oral bile acid replacement. Recently, cholic acid therapy has been found to be beneficial in suppressing hepatic function and minimizing nutritional deficiencies. However, there may be harmful effects in patients with significant hepatic dysfunction, and more research is needed to investigate the toxic effects of this therapy [3,5,15].

One of the limitations of this report is that not all patients had a pedigree done. Therefore, the history of consanguinity and prior history of metabolic disorders were unknown for two of our patients. Obtaining family pedigrees in the future may highlight any prior incidence or introduction of ZSD in the families of patients, as well as any history of consanguinity.

There is much to learn from the distinct Mixteco population present in Central California. The similarities between the cases in this region lead us to believe that a founder mutation is plausible. To support the theory that a founder mutation is present within the Mixteco community, screening patients from this population for single nucleotide polymorphisms is instrumental. Communicating these occurrences within the Mixteco population may also be important and allow parents to be more prepared when considering conception. Finding relevant ZSD cases from this population is essential in determining its incidence, and a larger-scale study could be conducted to determine this. It is essential to collaborate with other hospitals on the Central Coast to identify similar cases within this population. In addition, it would be useful to collaborate with researchers from Oaxaca, Mexico, to determine the incidence of ZSD in that region. Foremost, it is critical to highlight the importance of a pregnant patient’s Mixteco background in their social history and exercise high clinical suspicion for this disease in the fetus in order to best manage patients medically.

## Conclusions

Zellweger spectrum disorder is a group of autosomal recessive peroxisomal disorders caused by PEX gene mutations. These mutations lead to phenotypes with wide ranges in severity. In Central California, there have been various reports of the neonatal presentation of ZSD within the Mixteco population. This observation has been stipulated to be caused by a possible founder mutation. Here, we present three neonates with Zellweger syndrome of Mixteco descent. Consanguinity status was unknown; however, they all shared the same PEX6 gene mutation, c.1409G>C (p.Gly470Ala). With intentional neonatal screening and additional research, we hope that a founder mutation will be identified. This will allow Mixteco parents to make a more informed decision prior to conceiving, as well as aid physicians in exercising high clinical suspicion in pregnant Mixteco patients.
